# Impact of underweight on patients receiving curative-intent anticancer therapy: analysis of a large oncology database in Saudi Arabia

**DOI:** 10.1186/s12885-026-16499-w

**Published:** 2026-07-10

**Authors:** Emad Tashkandi

**Affiliations:** College of Medicine, Umm Al-Qura University, Makkah, 24242 Saudi Arabia

**Keywords:** Underweight, Cancer morbidity, Chemotherapy complications, Hospitalization, Oncology outcomes, Treatment delays

## Abstract

**Background:**

The clinical effects of underweight status on short-term treatment-related morbidity in patients receiving curative-intent anticancer therapy remain unclear.

**Methods:**

We conducted a retrospective observational cohort study using a comprehensive oncology database, including 2,504 consecutive patients with histologically confirmed malignancies treated between March 1 and June 30, 2020, at major cancer centers and outpatient clinics across Saudi Arabia. Patients who received curative-intent outpatient systemic anticancer therapy were eligible. Underweight (body mass index [BMI] < 18.5 kg/m²) and normal-weight patients were compared with respect to baseline characteristics, treatment patterns, and 30-day morbidity. Multivariable logistic regression was used to identify factors independently associated with morbidity.

**Results:**

In total, 787 patients met the eligibility criteria and were included in the analysis. Underweight patients were more frequently male (*P* < 0.001), whereas age (*P* = 0.764) and Eastern Cooperative Oncology Group (ECOG) performance status (*P* = 0.152) were similar between the BMI groups. Cancer stage distribution and curative treatment approach (neoadjuvant vs. adjuvant) did not differ significantly. Underweight patients were more likely to receive intravenous therapy (*P* = 0.005) and less likely to receive hormonal or targeted therapy. In unadjusted analyses, underweight patients had higher 30-day morbidity, with increased hospitalization (*P* = 0.023), emergency department visits (*P* = 0.034), and chemotherapy dose reductions (*P* = 0.004). After multivariable adjustment, underweight BMI was not independently associated with morbidity, whereas poorer ECOG performance status and treatment class were the strongest predictors. Intensive care unit admission rates did not differ between the groups.

**Conclusions:**

Underweight status was associated with increased unadjusted short-term treatment-related morbidity among patients receiving curative-intent anticancer therapy. However, this association was largely explained by functional status and treatment-related factors. These findings highlight the importance of early risk stratification and proactive supportive care rather than BMI alone to optimize treatment tolerance in this vulnerable population.

**Supplementary Information:**

The online version contains supplementary material available at https://doi.org/10.1186/s12885-026-16499-w.

## Background

The global cancer burden is projected to reach 28.4 million cases in 2040, a 47% increase from 2020 driven by demographic changes and a growing prevalence of modifiable risk factors [1]. In the Kingdom of Saudi Arabia, a steady improvement in survival has been observed over time for most cancers, which is comparable to that in Western countries [2].

Cancer is a complex disease influenced by multiple modifiable risk factors, including smoking, alcohol consumption, carcinogenic infectious agents, environmental exposure, physical inactivity, dietary patterns, air pollution, and an abnormal body mass index (BMI), encompassing both obesity and underweight [3]. The relationship between BMI and cancer outcomes has been extensively studied, with most attention focused on overweight and obesity owing to their well-documented association with greater overall mortality in patients with several malignancies [4]. Although underweight status (BMI < 18.5 kg/m²) has been associated with adverse long-term survival outcomes in several cancers, particularly in surgical oncology settings such as colorectal cancer [5, 6], evidence regarding short-term treatment-related morbidity in this context remains limited.

Cancer-related weight loss is characterized by the depletion of skeletal muscle mass with or without loss of adipose tissue and may represent an early clinical manifestation of malignancy, commonly reflecting underlying cancer cachexia [7]. Cancer-related weight loss results from pathophysiological derangements, including reduced food intake, physical inactivity, and metabolic alterations, such as increased energy expenditure, catabolism, and systemic inflammation [8, 9]. Patients with depleted resources and malnutrition experience reduced chemotherapeutic effects, increased adverse effects, treatment interruptions, shorter treatment times, increased surgical complications, and poor oncological outcomes [10, 11].

The effect of an underweight status on early treatment-related morbidity in patients receiving curative-intent anticancer therapies remains relatively underexplored. In Saudi Arabia, where the cancer burden and associated healthcare costs are increasing, an improved understanding of the factors influencing treatment outcomes is essential to inform national cancer control strategies [12]. Despite rising global obesity rates [13], a substantial proportion of patients with cancer remain underweight during treatment, a condition associated with impaired treatment tolerance and adverse outcomes [14]. Addressing these risks in this population may provide targeted supportive and nutritional interventions aimed at improving treatment delivery and short-term outcomes. The present study aimed to evaluate whether underweight status, relative to normal weight, was associated with 30-day morbidity among patients receiving curative-intent anticancer therapy using a large oncology database in Saudi Arabia.

## Methods

### Study design and population

This study included patients from a comprehensive oncology database representing multiple cancer centers and outpatient clinics in Saudi Arabia [15]. The source database comprised 2,504 consecutive patients with histologically confirmed malignancies who received anticancer therapy between March 1 and June 30, 2020.

We included patients treated with curative intent, including patients who were managed using curative-intent approaches, and excluded those treated with palliative intent. Treatment intent (curative vs. palliative) was determined based on the treatment oncology team’s documented intent, the regimen context recorded in the medical records, and the pharmacy treatment plan. Of the 2,504 patients in the source database, 787 met the eligibility criteria for curative-intent outpatient systemic therapy and were included in the final analysis. The remaining patients were excluded primarily due to palliative treatment intent, management with surgery or radiotherapy alone, receipt of best supportive care, or treatment limited to bone-modifying agents.

The Institutional Research Ethics Boards of the participating centers approved this study (IRB number 24-1328, November 24, 2024). Eligible patients completed at least one cycle of outpatient anticancer therapy. Additional details regarding the oncology database have been published previously [15]. A portion of the source database used in this study has been previously reported; however, the present analysis addressed a distinct research question with different eligibility criteria, exposure definitions, outcomes, and analytical approaches.

### Inclusion and exclusion criteria

Adult patients diagnosed with solid or hematological malignancies who received outpatient curative-intent anticancer therapy during the study period were eligible for inclusion. Patients under surveillance or follow-up only, those treated with curative surgery or radiotherapy alone, those receiving best supportive care, and those treated exclusively with bone-modifying agents (e.g., bisphosphonates or denosumab) were excluded. Missing data were minimal (< 1%), and patients with incomplete variables or limited follow-up were retained in the analysis when appropriate.

### Patient and treatment characteristics

Patient data included age, sex, and BMI. Clinical information included Eastern Cooperative Oncology Group performance status (ECOG-PS), cancer type, and cancer stage (I–IV). Treatment characteristics included the type of anticancer therapy (chemotherapy, immunotherapy, hormone therapy, or targeted therapy), route of administration (intravenous, subcutaneous, or oral), treatment setting (neoadjuvant or adjuvant), and number of treatment cycles.

### Study procedures

Electronic health records were reviewed by senior oncology physicians to confirm eligibility and to extract relevant data. Each patient’s data were anonymized by assigning a unique study code. Additional data collected included comorbidities, cancer diagnosis, type of treatment, and baseline BMI, classified according to established criteria: underweight (BMI < 18.5 kg/m²) and normal weight (BMI 18.5–24.9 kg/m²).

### Statistical analysis

Data were analyzed using IBM SPSS Statistics, version 25 (IBM Corp., Chicago, IL, USA). Continuous variables are presented as means with standard deviations and categorical variables as frequencies and percentages. Comparisons between the underweight and normal weight groups were performed using independent t-tests for continuous variables and chi-square tests for categorical variables, as appropriate.

Thirty-day morbidity was defined as a composite binary outcome comprising hospitalization, emergency department visits, chemotherapy delay or dose reduction, or intensive care unit admission (ICU) within 30 days of systemic anticancer therapy. The composite outcome includes events of varying clinical severity, ranging from chemotherapy dose modifications to more severe events such as ICU admission, and should be interpreted accordingly. Unadjusted associations between baseline demographic, clinical, tumor-related, and treatment-related variables and 30-day morbidity were explored.

To identify factors independently associated with 30-day morbidity, a multivariate logistic regression model was constructed. The covariates entered into the model were selected a priori based on clinical relevance and included age, sex, BMI category (underweight vs. normal weight), ECOG-PS, cancer stage, treatment class, treatment intent (neoadjuvant vs. adjuvant), and route of administration. Results are reported as odds ratios (ORs) with corresponding 95% confidence intervals (CIs).

Because no variable had more than 10% missing data, we conducted a complete case analysis. All statistical tests were two-sided, and a *P*-value < 0.05 was considered statistically significant. This study is reported in line with the Strengthening the Reporting of Observational Studies in Epidemiology (STROBE) recommendations for cohort studies [16].

## Results

### Baseline demographic and clinical characteristics

Data were collected from 787 patients. Patient demographic and clinical characteristics at initial diagnosis are shown in Table 1. The prevalence of being underweight did not differ by age category. Regarding sex, a significant difference was observed (*P* < 0.001), with 57.7% of underweight patients being men, compared with 41.8% being women. Underweight status was observed in 46.2% of patients with ECOG-PS 0–1 and 62.5% of those with ECOG-PS > 1, although the difference was not statistically significant (*P* = 0.152).

**Table 1:** Baseline demographic and clinical characteristics

Patient characteristics	All patients (*n* = 787)	Underweight (*n* = 367, 46.6%)	Normal weight (*n* = 420, 53.4%)	*P*-value
Age, *n* (%)				
>65 years	117 (14.9)	53 (45.3)	64 (54.7)	0.764
≤65 years	670 (85.1)	314 (46.9)	356 (53.1)	
Sex, *n* (%)				
Male	239 (30.4)	138 (57.7)	101 (42.3)	< 0.001
Female	548 (69.6)	229 (41.8)	319 (58.2)	
ECOG, *n* (%)				
0–1	770 (98.0)	356 (46.2)	414 (53.8)	0.152
>1	16 (2.0)	10 (62.5)	6 (37.5)	
Stage, *n* (%)				
I-II	280 (36.6)	125 (44.6)	155 (55.4)	0.340
III	345 (45.2)	156 (45.2)	189 (54.8)	
IV	139 (18.2)	72 (51.8)	67 (48.2)	
Type of therapy, *n* (%)				
Chemotherapy	506 (64.4)	256 (50.6)	250 (49.4)	0.05
Immunotherapy	7 (0.9)	5 (71.4)	2 (28.6)	
Hormonal therapy	165 (21.0)	62 (37.6)	103 (62.4)	
Targeted therapy	93 (11.8)	40 (43.0)	53 (57.0)	
Route, *n* (%)				
Intravenous	551 (70)	278 (50.5)	273 (49.5)	0.005
Oral	203 (25.8)	77 (37.9)	126 (62.1)	
Subcutaneous	33 (4.2)	12 (36.4)	21 (63.6)	
Type of curative treatment, *n* (%)				
Neoadjuvant	161 (26.5)	74 (46.0)	87 (54.0)	0.308
Adjuvant	446 (73.5)	184 (41.3)	262 (58.7)	
Number of treatment cycles, (mean ± SD)	4.73 ± 5.77			

Values are presented as numbers (%) unless otherwise noted. Statistical significance was set at *P* < 0.05

Patients not classified under neoadjuvant or adjuvant categories include those receiving concurrent chemoradiation or systemic therapy without a clear neoadjuvant/adjuvant designation

*BMI *body mass index, *ECOG-PS *Eastern Cooperative Oncology Group performance status, *SC *subcutaneous, *SD *standard deviation

The proportion of underweight patients was similar across cancer stages: 44.6% in stage I–II disease, 45.2% in stage III, and 51.8% in stage IV. No statistically significant difference in cancer stage distribution according to BMI was observed (*P* = 0.340).

Regarding the type of therapy, the distribution of chemotherapy use was similar between underweight and normal-weight patients (50.6% vs. 49.4%, *P* = 0.05). Hormonal and targeted therapies were more frequently administered to normal-weight patients, although these differences were not statistically significant.

Regarding the route of therapy, underweight status was more common among patients receiving intravenous treatment, with 50.5% classified as underweight, compared with 37.9% of those receiving oral therapy and 36.4% receiving subcutaneous therapy. This distribution differed significantly according to BMI category (*P* = 0.005).

Finally, 46% of underweight patients received neoadjuvant therapy for curative treatment, whereas 41.3% received adjuvant therapy, with no significant difference (*P* = 0.308). The mean number of treatment cycles in the overall cohort was 4.73 ± 5.77 and was similar between the two groups.

## Cancer diagnoses across different BMI groups

The distribution of cancer diagnoses according to BMI category is shown in Table 2. The most common malignancies in the overall cohort were breast cancer (48.8%), lung cancer (13.3%), and lymphoma (13.0%). Among patients with lymphoma, 53.9% were underweight, compared with 44.8% of patients with lung cancer and 38.0% of those with breast cancer.

**Table 2:** Cancer diagnoses across different BMI groups

Cancer diagnosis	All patients	Underweight	Normal weight	*P*-value
Breast	384 (48.8)	146 (38.0)	238 (62.0)	0.457
Lymphoma	102 (13.0)	55 (53.9)	47 (46.1)	0.624
Lung	105 (13.3)	47 (44.8)	58 (55.2)	0.132
Colorectal	12 (1.5)	9 (75.0)	3 (25.0)	0.513
Gynecological	34 (4.3)	23 (67.6)	11 (32.4)	0.146
Head and neck	45 (5.7)	22 (48.9)	23 (51.1)	0.632
Sarcoma	39 (5.0)	25 (64.1)	14 (35.9)	0.542
Other	66 (8.4)	37 (56.1)	29 (43.9)	0.054

Values are presented as numbers (%) unless otherwise noted

*BMI* Body mass index

A higher proportion of underweight patients was observed in less common cancer types, including colorectal cancer (75.0%, 9 of 12 patients), gynecological cancers (67.6%), and sarcoma (64.1%). However, these findings should be interpreted with caution given the small sample sizes of these subgroups. Overall, no statistically significant differences in the cancer diagnosis distribution according to BMI category were observed.

### Summary of 30-day morbidity

Table 3 shows the impact of patient weight on 30-day morbidity following anticancer treatment. Overall, 156 of the 787 patients (19.8%) experienced at least one morbidity event within 30 days of anticancer therapy. Among these patients with 30-day morbidity, 59.0% were underweight and 41.0% were normal weight (*P* = 0.001).

**Table 3:** Summary of 30-day morbidity

Variable	All patients	Underweight BMI	Normal weight BMI	*P*-value
30-day morbidity (yes)	156	92 (59.0)	64 (41.0)	0.001
Cause of 30-day morbidity				
Hospitalization	84	49 (58.3)	35 (41.7)	0.023
ER visits	97	55 (56.7)	42 (43.3)	0.034
Delay of chemotherapy	73	41 (56.2)	32 (43.8)	0.050
Dose reduction	39	27 (69.2)	12 (30.8)	0.004
ICU admission	17	8 (47.1)	9 (52.9)	0.546

Values are presented as numbers (%) unless otherwise noted. The primary outcome was 30-day morbidity, defined as morbidity within 30 days of the last anticancer treatment cycle, which included any of the following: hospitalization, emergency room (ER) visit, delay in chemotherapy or dose reduction, and intensive care unit (ICU) admission. Statistical significance was set at *P* < 0.05

Hospitalization occurred in 84 patients (10.7%), affecting 58.3% of underweight patients and 41.7% of normal-weight patients (*P* = 0.023). Emergency department visits were reported in 97 patients (12.3%), of whom 56.7% were underweight and 43.3% were normal weight (*P* = 0.034). Chemotherapy delays occurred in 73 patients (9.3%), with a higher proportion of underweight patients (56.2% vs. 43.8%); however, this difference was of borderline statistical significance (*P* = 0.050). Chemotherapy dose reductions were observed in 39 patients (5.0%) and were significantly more frequent in underweight patients (69.2%) than in normal-weight patients (30.8%) (*P* = 0.004).

ICU admission was uncommon (17 patients, 2.2%) and did not differ significantly according to BMI category, with 47.1% of admissions occurring in underweight patients and 52.9% in normal-weight patients (*P* = 0.546).

### Factors associated with 30-day morbidity

A multivariable logistic regression model was fitted to identify factors independently associated with 30-day morbidity following curative-intent anticancer therapy, as shown Table 4. After adjustment for all covariates, underweight BMI was not significantly associated with 30-day morbidity compared with normal BMI (OR = 1.32, 95% CI 0.91–1.91; *P* = 0.143).

**Table 4:** Multivariable logistic regression analysis of factors associated with 30-day morbidity

Variable	OR	95% CI	*P*-value
Intercept	0.24	0.15–0.39	< 0.005
Body mass index (BMI)			
Normal weight	Reference	—	—
Underweight	1.32	0.91–1.90	0.143
Age (years)			
≤65	Reference	—	—
>65	1.05	0.71–1.55	0.807
Sex			
Male	Reference	—	—
Female	0.80	0.59–1.21	0.158
Treatment class			
Chemotherapy	Reference	—	—
Hormonal therapy	0.12	0.05–0.26	< 0.001
Immunotherapy	0.48	0.22–0.97	0.047
Targeted therapy	0.32	0.17–0.53	< 0.001
ECOG = PS	2.55	1.84–3.55	< 0.001
Route of administration			
Intravenous	Reference	—	—
Oral	1.34	0.82–2.19	0.239
Subcutaneous	0.93	0.24–2.80	0.903

Outcomes: Any 30-day morbidity (yes/no) was defined as hospitalization, emergency department visit, chemotherapy delay or dose reduction, or intensive care unit admission within 30 days of anticancer therapy

The multivariate logistic regression model was adjusted for age, sex, BMI category, ECOG-PS, cancer stage, treatment class, treatment intent, and route of administration

Statistical significance was defined as a two-sided *P*-value < 0.05

*BMI* Body mass index, *CI* Confidence interval, *ECOG-PS* Eastern Cooperative Oncology Group performance status, *OR* Odds ratio

Similarly, age > 65 years was not associated with morbidity (OR = 1.05, 95% CI 0.71–1.55; *P* = 0.807), and female sex was not significantly associated with morbidity compared with male sex (OR = 0.80, 95% CI 0.59–1.09; *P* = 0.158). Compared with chemotherapy, hormonal therapy (OR = 0.12, 95% CI 0.05–0.27; *P* < 0.001), immunotherapy (OR = 0.48, 95% CI 0.22–0.97; *P* = 0.047), and targeted therapy (OR = 0.32, 95% CI 0.18–0.54; *P* < 0.001) were associated with significantly lower odds of 30-day morbidity. The association observed with immunotherapy should be interpreted with caution given the small subgroup size (*n* = 7). In contrast, higher ECOG-PS was strongly associated with higher odds of morbidity (OR = 2.55 per one-unit increase, 95% CI 1.84–3.55; *P* < 0.001). The route of administration was not significantly associated with morbidity (oral vs. intravenous: OR = 1.34, 95% CI 0.82–2.19; *P* = 0.239; subcutaneous vs. intravenous: OR = 0.93, 95% CI 0.25–2.80; *P* = 0.903).

## Discussion

In this large multicenter cohort of patients receiving curative-intent systemic anticancer therapy, underweight status was more frequently observed among men than women, consistent with prior population-based analyses that demonstrated sex-specific differences in BMI distribution and body composition [17]. Importantly, the baseline functional status, as measured by the ECOG-PS score, did not differ significantly between underweight and normal-weight patients, indicating a broadly comparable pretreatment performance in this selected curative-intent cohort.

The cancer stage at diagnosis was similarly distributed across BMI categories, with underweight patients representing early, locally advanced, and metastatic stages. The absence of a significant association between BMI and disease stage aligns with findings from smaller cohorts [18] and suggests that underweight status in this setting is not merely a surrogate for more advanced disease. This distinction is clinically relevant because it indicates that differences in short-term outcomes are unlikely to be driven by stage imbalance alone.

With respect to treatment patterns, chemotherapy use was comparable between underweight and normal-weight patients, suggesting that BMI does not substantially influence initial eligibility for chemotherapy in routine clinical practice. However, hormonal and targeted therapies were administered less frequently to underweight patients, which is a pattern previously observed in large pan-cancer cohorts [19]. These differences may reflect clinical concerns regarding treatment tolerance, anticipated duration of therapy, or functional reserve, rather than formal contraindications. Prior work has demonstrated that underweight patients are more likely to receive fewer chemotherapy cycles and to discontinue treatment early [20], suggesting that BMI may influence treatment intensity over time rather than initial treatment selection.

Data regarding the influence of BMI on the route of systemic therapy are limited. In the present study, underweight patients were significantly more likely to receive intravenous rather than oral or subcutaneous administration. Population-level data from England have consistently shown that weight loss during systemic anticancer therapy is independently associated with treatment modifications across multiple tumor types, supporting nutritional vulnerability as an important determinant of treatment delivery [21]. This likely reflects practical considerations including impaired oral intake, concerns regarding gastrointestinal absorption, and reduced subcutaneous tissue available for drug delivery in underweight individuals. Reduced muscle mass and subcutaneous fat in underweight patients affects drug absorption and distribution [22]. Collectively, these findings suggest that low body weight may influence route selection and treatment modification through impaired treatment tolerance and altered pharmacokinetics, rather than disease-related factors alone.

Underweight status did not significantly influence the choice between neoadjuvant and adjuvant treatment approaches, with similar proportions of underweight patients receiving each modality. This finding suggests that within a curative-intent framework and with appropriate supportive care, underweight patients can successfully undergo either treatment strategy. Although data specifically addressing malnutrition in this context are limited, these results align with growing evidence from the Eastern Mediterranean Region, emphasizing the importance of context-specific, multidisciplinary cancer care that integrates nutritional and supportive interventions to optimize treatment delivery and outcomes irrespective of BMI [23].

The distribution of cancer diagnoses in this cohort reflected the composition of the underlying database, with breast, lymphoma, and lung cancers being the most common. Among underweight patients, higher proportions of lymphoma, colorectal, and gynecological cancers were observed. This pattern differs from population-based data from the United Kingdom, where underweight status was more strongly associated with lung and upper gastrointestinal cancers, largely driven by smoking-related risk [24]. As smoking status was not captured in the present study, direct comparisons were limited. No statistically significant differences in cancer type distribution by BMI were observed, indicating that underweight status in this cohort was not confined to specific malignancies.

In the unadjusted analyses, underweight patients experienced significantly higher 30-day morbidity rates, including increased hospitalizations and emergency department visits, following curative-intent therapy. These findings are consistent with those of previous studies identifying underweight status as a marker of vulnerability and worse short-term outcomes in cancer populations [25, 26]. Higher rates of acute-care utilization likely reflect reduced physiological reserves, increased treatment toxicity, and subclinical frailty.

However, after multivariable adjustment, underweight BMI was no longer independently associated with 30-day morbidity. Instead, the ECOG-PS emerged as the strongest predictor, with each one-unit worsening associated with more than a two-fold increase in morbidity risk. This finding underscores functional reserve as the dominant determinant of early treatment tolerance, superseding BMI. Notably, ECOG-PS correlates with sarcopenia, a condition characterized by reduced skeletal muscle mass and impaired physiological resilience, supporting its role as an integrative marker of vulnerability beyond BMI alone [27]. Therefore, underweight status appears to function primarily as a clinical marker of vulnerability rather than an independent causal driver of short-term morbidity.

Treatment class was independently associated with 30-day morbidity. Hormonal therapy, immunotherapy, and targeted therapy were associated with significantly lower morbidity than chemotherapy, consistent with outpatient data linking non-chemotherapy regimens to better nutritional status and lower malnutrition risk [28]. The observed association with immunotherapy should be interpreted with caution given the small sample size of this subgroup, which may limit the precision and generalizability of the estimate. This finding supports treatment-related toxicity and functional reserve as the principal drivers of early morbidity and reinforces the biological plausibility of the adjusted model.

Although chemotherapy delays were more frequent in underweight patients, the borderline statistical significance suggests that this association should be interpreted with caution. In contrast, chemotherapy dose reductions were significantly more common in underweight patients, which is consistent with the findings of previous systematic reviews [29]. These findings reflect reduced treatment tolerance and underscore the importance of individualized dosing strategies, early nutritional assessment, and proactive supportive care.

ICU admissions did not differ significantly between BMI groups, suggesting that although underweight patients experience higher rates of treatment-related morbidity, they are not at an increased risk of severe, life-threatening complications requiring ICU admission. ICU decision-making in oncology remains multifactorial and is influenced by performance status, comorbidity burden, prognosis, and treatment intent, rather than by nutritional status alone [30].

The strengths of this study include its large, multicenter, real-world design; the inclusion of a substantial number of underweight patients; and its focus on short-term, clinically meaningful outcomes in a curative-intent setting. This study addresses a clear gap in the literature, as underweight patients are frequently underrepresented in oncology research and randomized trials.

This study has several limitations. The observational design precludes causal inferences, and residual confounding factors cannot be excluded. The predominance of patients with ECOG-PS 0–1 (98%) reflects the curative-intent nature of the cohort and may introduce a ceiling effect, potentially limiting the ability to detect associations between performance status and outcomes. The study period coincided with the early phase of the COVID-19 pandemic, which may have influenced healthcare utilization patterns, including emergency department visits and hospitalization rates. A single-country setting may limit generalizability, and detailed nutritional metrics, smoking history, and socioeconomic factors were not comprehensively captured. The follow-up was limited to short-term outcomes, precluding assessment of long-term survival or functional recovery. The distribution of cancer diagnoses reflects the study design and cohort composition, which was restricted to patients who received outpatient systemic anticancer therapy. Consequently, certain malignancies, including colorectal cancer, were underrepresented, and cancer-type–specific findings should be interpreted descriptively and with caution. Although the source database has been used in previous publications, the present analysis addresses a conceptually and analytically distinct research question, focusing on underweight status and early treatment-related morbidity in a curative-intent setting, thereby minimizing redundancy.

Further research is needed to elucidate the biological and clinical mechanisms underlying reduced treatment tolerance in underweight patients, and to identify strategies that optimize treatment delivery while minimizing toxicity. Our findings support the importance of a multidisciplinary approach that incorporates early nutritional support, physical conditioning, and psychosocial care to mitigate treatment-related morbidity. Data on nutritional interventions were not systematically captured in the database, which limits the ability to directly link observed outcomes with nutritional management strategies. Importantly, underweight status alone should not be viewed as a contraindication to curative-intent therapy; rather, it should prompt proactive supportive interventions aimed at maintaining treatment intensity when clinically appropriate. Prospective studies focusing specifically on underweight populations are warranted to inform evidence-based guidelines and standardize management strategies.

In summary, underweight status was associated with increased unadjusted short-term morbidity in patients receiving curative-intent anticancer therapy; however, this association was largely explained by performance status and treatment-related factors in the adjusted analyses. These findings emphasize that underweight status should not be viewed as a contraindication to curative therapy but rather as a signal for heightened clinical vigilance and proactive supportive care. A multidisciplinary approach incorporating early nutritional assessment, physical conditioning, and individualized treatment planning is essential to optimize the outcomes in this vulnerable population.

## Supplementary Information

Supplementary Material 1.

## Data Availability

The datasets generated and analyzed in the current study are available upon request.

## Abbreviations

BMI: Body mass index
CI: Confidence interval
ECOG: Eastern Cooperative Oncology Group
ECOG-PS: Eastern Cooperative Oncology Group performance status
ICU: Intensive care unit
OR: Odds ratio
STROBE: Strengthening the Reporting of Observational Studies in Epidemiology
