# Supplementary material for: Impact of underweight on patients receiving curative-intent anticancer therapy: analysis of a large oncology database in Saudi Arabia
Source: BMC Cancer. 2026 Jul 10;26:1196. doi: 10.1186/s12885-026-16499-w (PMC13640195; doi:10.1186/s12885-026-16499-w)
Supplement: Supplementary file 1 [file 12885_2026_16499_MOESM1_ESM.docx]

**Table 1** Baseline demographic and clinical characteristics

| **Patient characteristics** | **All patients (*n* = 787)** | **Underweight (*n* = 367, 46.6%)** | **Normal weight (*n* = 420, 53.4%)** | ***P*-value** |
| --- | --- | --- | --- | --- |
| Age, *n* (%) |  |  |  |  |
| >65 years | 117 (14.9) | 53 (45.3) | 64 (54.7) | 0.764 |
| ≤65 years | 670 (85.1) | 314 (46.9) | 356 (53.1) |  |
| Sex, *n* (%) |  |  |  |  |
| Male | 239 (30.4) | 138 (57.7) | 101 (42.3) | <0.001 |
| Female | 548 (69.6) | 229 (41.8) | 319 (58.2) |  |
| ECOG, *n* (%) |  |  |  |  |
| 0-1 | 770 (98.0) | 356 (46.2) | 414 (53.8) | 0.152 |
| >1 | 16 (2.0) | 10 (62.5) | 6 (37.5) |  |
| Stage, *n* (%) |  |  |  |  |
| I-II | 280 (36.6) | 125 (44.6) | 155 (55.4) | 0.340 |
| III | 345 (45.2) | 156 (45.2) | 189 (54.8) |  |
| IV | 139 (18.2) | 72 (51.8) | 67 (48.2) |  |
| Type of therapy, *n* (%) |  |  |  |  |
| Chemotherapy | 506 (64.4) | 256 (50.6) | 250 (49.4) | 0.05 |
| Immunotherapy | 7 (0.9) | 5 (71.4) | 2 (28.6) |  |
| Hormonal therapy | 165 (13.4) | 62 (37.6) | 103 (62.4) |  |
| Targeted therapy | 93 (11.8) | 40 (43.0) | 53 (57.0) |  |
| Route, *n* (%) |  |  |  |  |
| Intravenous | 551 (70) | 278 (50.5) | 273 (49.5) | 0.005 |
| Oral | 203 (25.8) | 77 (37.9) | 126 (62.1) |  |
| Subcutaneous | 33 (4.2) | 12 (36.4) | 21 (63.6) |  |
| Type of curative treatment, *n* (%) |  |  |  |  |
| Neoadjuvant | 161 (26.5) | 74 (46.0) | 87 (54.0) | 0.308 |
| Adjuvant | 446 (73.5) | 184 (41.3) | 262 (58.7) |  |
| Number of treatment cycles, (mean ± SD) | 4.73±5.77 |  |  |  |

Values are presented as numbers (%) unless otherwise noted. Statistical significance was set at *P* < 0.05.

*BMI* Body mass index, *ECOG-PS* Eastern Cooperative Oncology Group performance status, *SC* Subcutaneous, *SD* Standard deviation

**Table 2** Cancer diagnoses across different BMI groups

| **Cancer diagnosis** | **All patients** | **Underweight** | **Normal weight** | ***P*-value** |
| --- | --- | --- | --- | --- |
| Breast | 384 (48.8) | 146 (38.0) | 238 (62.0) | 0.457 |
| Lymphoma | 102 (13.0) | 55 (53.9) | 47 (46.1) | 0.624 |
| Lung | 105 (13.3) | 47 (44.8) | 58 (55.2) | 0.132 |
| Colorectal | 12 (1.5) | 9 (75.0) | 3 (25.0) | 0.513 |
| Gynecological | 34 (4.3) | 23 (67.6) | 11 (32.4) | 0.146 |
| Head and neck | 45 (5.7) | 22 (48.9) | 23 (51.1) | 0.632 |
| Sarcoma | 39 (5.0) | 25 (64.1) | 14 (35.9) | 0.542 |
| Other | 66 (8.4) | 37 (56.1) | 29 (43.9) | 0.054 |

Values are presented as numbers (%) unless otherwise noted.

*BMI* Body mass index

**Table 3** Summary of 30‑day morbidity

| **Variable** | **All patients** | **Underweight BMI** | **Normal weight BMI** | ***P*-value** |
| --- | --- | --- | --- | --- |
| 30-day morbidity (yes) | 156 | 92 (59.0) | 64 (41.0) | 0.001 |
| **Cause of 30‑day morbidity** | | | | |
| Hospitalization | 84 | 49 (58.3) | 35 (41.7) | 0.023 |
| ER visits | 97 | 55 (56.7) | 42 (43.3) | 0.034 |
| Delay of chemotherapy | 73 | 41 (56.2) | 32 (43.8) | 0.050 |
| Dose reduction | 39 | 27 (69.2) | 12 (30.8) | 0.004 |
| ICU admission | 17 | 8 (47.1) | 9 (52.9) | 0.546 |

Values are presented as numbers (%) unless otherwise noted. The primary outcome was 30-day morbidity, defined as morbidity within 30 days of the last anticancer treatment cycle, which included any of the following: hospitalization, emergency room (ER) visit, delay in chemotherapy or dose reduction, and intensive care unit (ICU) admission. Statistical significance was set at *P* < 0.05.

**Table 4** Multivariable logistic regression analysis of factors associated with 30-day morbidity

| **Variable** | **OR** | **95% CI** | ***P*-value** |
| --- | --- | --- | --- |
| Intercept | 0.24 | 0.15–0.39 | <0.005 |
| Body mass index (BMI) |  |  |  |
| Normal weight | Reference | — | — |
| Underweight | 1.32 | 0.91–1.90 | 0.143 |
| Age (years) |  |  |  |
| ≤65 | Reference | — | — |
| >65 | 1.05 | 0.71–1.55 | 0.807 |
| Sex |  |  |  |
| Male | Reference | — | — |
| Female | 0.80 | 0.59–1.21 | 0.158 |
| Treatment class |  |  |  |
| Chemotherapy | Reference | — | — |
| Hormonal therapy | 0.12 | 0.05–0.26 | <0.001 |
| Immunotherapy | 0.48 | 0.22–0.97 | 0.047 |
| Targeted therapy | 0.32 | 0.17–0.53 | <0.001 |
| ECOG=PS | 2.55 | 1.84–3.55 | <0.001 |
| Route of administration |  |  |  |
| Intravenous | Reference | — | — |
| Oral | 1.34 | 0.82–2.19 | 0.239 |
| Subcutaneous | 0.93 | 0.24–2.80 | 0.903 |

Outcomes: Any 30-day morbidity (yes/no) was defined as hospitalization, emergency department visit, chemotherapy delay or dose reduction, or intensive care unit admission within 30 days of anticancer therapy.

The multivariate logistic regression model was adjusted for age, sex, BMI category, ECOG-PS, cancer stage, treatment class, treatment intent, and route of administration.

Statistical significance was defined as a two-sided *P*-value <0.05.

*BMI* Body mass index, *CI* Confidence interval, *ECOG-PS* Eastern Cooperative Oncology Group performance status, *OR* Odds ratio
